# Editorial: Effects of Membrane Lipids on Protein Function

**DOI:** 10.3389/fcell.2021.675264

**Published:** 2021-04-29

**Authors:** Rutilio A. Fratti

**Affiliations:** ^1^Department of Biochemistry, University of Illinois at Urbana-Champaign, Urbana, IL, United States; ^2^Center for Biophysics and Quantitative Biology, University of Illinois at Urbana-Champaign, Urbana, IL, United States

**Keywords:** glycolipid, sphingolipid, phosphoinositide, cholesterol, phosphatidic acid, diacylglycerol, membrane contact sites, phospholipid transfer protein

Eukaryotes are compartmentalized into membrane bound organelles that are separated by the cytosol, yet communication between organelles is required for cellular homeostasis and for responding to outside signals. The transfer of information between organelles occurs through multiple mechanisms. For instance, the binding of ligands to their receptors transduces signals across the membrane bilayer to activate signal transduction pathways. Information transfer also occurs through vesicular trafficking and the movement of cargo between organelles; the secretion of soluble factors into the extracellular space; and the uptake of material from outside the cell for degradation *via* the endolysosomal pathway. While proteins drive these pathways, the composition of the membrane can have profound effects on regulating their efficacy. Dysregulation of the mechanics that affect membrane composition can lead to a plethora of maladies (e.g., cancer) that are manifest through defective protein function. Thus, it is important to understand the how lipids fundamentally affect such pathways.

For the most part, organellar membranes are composed of amphipathic lipids that assemble into bilayers with the polar head groups facing the cytosol or extracellular environment, while the hydrophobic groups face each other to exclude water. The lipid constituents of membranes fall into three major groups classified as glycerophospholipids (GPL), sphingolipids (SL), and sterols. GPLs contain a glycerol backbone with fatty acids attached as esters at two carbons while the third hydroxyl is unmodified or linked to a head group through a phosphate ester linkage. GPLs include the bulk lipids phosphatidylcholine (PC), phosphatidylethanolamine (PE), and phosphatidylserine (PS), as well as lipids that are at low concentrations such as phosphatidic acid (PA), diacylglycerol (DAG), and phosphoinositides (PI). SLs contain a sphingoid base backbone that includes the amino alcohol sphingosine. This sphingosine backbone can be linked to a fatty acid through an amide bond. SLs include Ceramide (Cer), Sphingomyelin (SM), and glycosylated sphingolipids. The lipid composition of organelles differs widely to give each compartment a signature profile that affects the function of membrane-associated proteins.

The lipid bilayer imposes two major influences on membrane proteins. These effects can be distinguished as specific protein-lipid interactions and non-specific interactions that are influenced by the physical properties of membranes. Lipids can recruit soluble proteins that contain lipid binding domains that recognize a particular lipid species. Such domains are conserved modular protein folds including the PH (pleckstrin homology) (Harlan et al., 1994), PX (phox homology) (Xu et al., 2001) and FYVE (Fab1p, YOTB, Vac1p, and EEA1) (Gillooly et al., 2000) domains that recognize specific phosphorylated forms of PIs, e.g., phosphatidylinositol 3-phosphate (PI3P). Lipids can also be bound by protein regions lacking a conserved protein fold including polybasic regions that bind anionic lipids such as the MARCKS effector domain (MED) that preferentially binds PI(4,5)P_2_ (Denisov et al., 1998). Here, Ueda et al. show that the hypervariable region of the small GTPases Rab4 and Rab5 promote the intrinsic tethering capability of these Rabs when PS, PI, and cholesterol are present.

While organelles have distinct sets of lipids, their ratio is not static. Instead, the local composition of membranes is under constant remodeling as part of regulating cellular functions. For instance, phosphoinositides can be differentially phosphorylated and dephosphorylated by specific kinases and phosphatases. Such changes not only affect the recruitment of proteins with specific lipid binding domains but alter the local surface charge of a membrane. Lipids can also be modified through the action of phospholipase C (PLC) and PLD to remove lipid head groups to make DAG and PA that alter protein binding and function as seen by the activation of protein kinase C by DAG (Kong et al., 1991) or the inhibition of SNARE activation through the sequestration of Sec18 by PA (Starr et al., 2019). Lipids can also be modified through the removal of an acyl chain by PLA1, PLA2, and PLB to make lysolipids, which engage specific receptors (Moolenaar, 2000) and affect membrane curvature (Kooijman et al., 2003). The removal of acyl changes can also be coupled with their exchange through the action of acyltransferases (Sanford and Frosolono, 1983).

Not only does the overall lipid composition of a membrane affect protein function, but the distribution of lipids across the bilayer adds a level of complexity to the interaction between proteins and membranes. Changing the characteristics of a leaflet can occur through translocating specific lipids across the bilayer through the function of flippases (Backer and Dawidowicz, 1987) (Fazeli et al.) and floppases (Dekkers et al., 1998) to establish lipid asymmetry between leaflets. In contrast, lipid asymmetry can be homogenized through the indiscriminate translocation of lipids by scramblases (Daleke, 2003). Other modifications occur through non-vesicular lipid transfer between membranes (Bankaitis et al., 1990). Finally, the physical characteristics of a membrane can change through the formation of sterol and sphingolipid rich membrane microdomains that both thicken and stiffen membranes to affect protein function (Edidin, 2003; Wang and Silvius, 2003). These modifications along with lipid interdigitation, hydrophobic mismatching, and membrane compression can exquisitely regulate protein function and their pathways (Andersen and Koeppe, 2007). Finally, various physical properties of membranes can be altered by small molecules including those that affect the electrical potential of membranes (Efimova et al.) or by increasing lipid disorder to potentiate the effects of antifungal drugs (Zakharova et al.).

In this collection of papers, we see a sample of how the membrane can affect protein function. Starting with phosphoinositides, the included papers show how these lipids affect the acidification of organelles (Banerjee and Kane), protein trafficking in cilia (Nechipurenko), recruitment of RUFY effectors to endosomes (Char and Pierre) and establishing membrane contact sites (Zaman et al.). As mentioned above, PIs can be modified by phospholipases to produce PA and DAG. Here, Moon and Jun show how mitochondrial fusion is regulated by PA. Another paper shows that converting PA to lysoPA through PLA activity controls neurite outgrowth (Maemoto et al.). Finally, DAG-rich structures are shown to affect lipid droplet consumption by tubular endoplasmic reticulum (Ganesan et al.).

Other lipid classes represented in this collection include cholesterol, glycolipids, and sphingolipids. In yeast, ergosterol is shown to affect the GTPase Sey1/atlastin during the fusion of endoplasmic reticulum microsomes (Moon and Jun). Glycolipids include GPLs and SLs where the head group has been modified with carbohydrates. Here Hanafusa et al., show how the two glycolipids lactosylceramide and phosphatidylglucoside differentially regulate separate classes of lipid rafts at the plasma membrane. Finally, Hurst and Fratti discuss how ergosterol and sphingolipids affect vacuole fusion.

## Open Questions and Challenges

The regulation of proteins by membrane lipids is a rapidly expanding and exciting area of research. While great advances have been made in recent years, there are many questions that remain elusive and challenging to address. Outstanding questions include:

- How do different lipids act in concert to affect specific protein-lipid interactions? Membrane proteins are surrounded by a cocktail of lipids that change in a spatiotemporal manner. In other words, the stoichiometry of lipids surrounding a particular protein or set of proteins (e.g., SNAREs) will change through the duration of the pathway. Lipids such as phosphoinositides and their metabolites are rapidly modified through the actions of specific kinases, phosphatases and lipases. Thus, dysregulating the timing and order of lipid modification can have profound effects on the function of a protein and block the progression of a pathway.
- How can lipid dynamics be tracked by fluorescence microscopy without altering said dynamics or their inherent physical properties? Lipids are commonly tracked with fluorescent probes that recognize specific lipid head groups. While informative, the use of bulky probes can block lipid interactions and alter their lateral diffusion. These probes could also have off target binding and skew the readout. Alternatively, an acyl chain is replaced with a fluorophore. Because the physical properties of acyl chains partially or wholly define the biochemical behavior of a lipid, replacing one with a fluorophore essentially creates a new lipid whose properties are irrelevant and can introduce unwanted physical effects to the system of interest.
- How do raft-like lipid microdomains and membrane asymmetry affect protein function on endomembranes? Much of the work on membrane microdomains and asymmetry has been done on the plasma membrane, thus their importance within the cell is less understood.
- How do we best determine the true result of altering lipid modification? The study of lipid modification on protein function is commonly tested by inhibiting the modifying enzyme. The resulting phenotype is a mixture of blocking the production of a new lipid with the accumulation of the precursor. While this can be partially addressed using defined reconstituted systems, we have to consider that the missing lipid has to be replaced with another other lipid further altering lipid stoichiometry and introducing a new variable.
- How do you measure the real-time effects of dynamic changes in hydrophobic mismatch and interdigitation on protein function? Changes in the thickness of membranes through such mechanisms can have dramatic effects on protein organization and function. Yet, the rapid lateral remodeling of lipids and transient changes in bilayer thickness is difficult to track.

## Author Contributions

RF wrote the editorial.

## Conflict of Interest

The author declares that the research was conducted in the absence of any commercial or financial relationships that could be construed as a potential conflict of interest.
